# Gender specific click and tone burst evoked ABR datasets from mice lacking the Ca_v_2.3 R-type voltage-gated calcium channel

**DOI:** 10.1016/j.dib.2018.10.056

**Published:** 2018-10-24

**Authors:** Andreas Lundt, Christina Henseler, Carola Wormuth, Julien Soos, Robin Seidel, Ralf Müller, Muhammad Imran Arshaad, Karl Broich, Jürgen Hescheler, Agapios Sachinidis, Dan Ehninger, Anna Papazoglou, Marco Weiergräber

**Affiliations:** a Experimental Neuropsychopharmacology, Federal Institute for Drugs and Medical Devices (Bundesinstitut für Arzneimittel und Medizinprodukte, BfArM), Kurt-Georg-Kiesinger-Allee 3, 53175 Bonn, Germany; b Cognitive Neurophysiology, Department of Psychiatry and Psychotherapy, University of Cologne, Faculty of Medicine, Kerpener Str. 62, 50937 Cologne, Germany; c Institute of Neurophysiology, University of Cologne, Faculty of Medicine, Robert-Koch-Str. 39, 50931 Cologne, Germany; d Molecular and Cellular Cognition, German Center for Neurodegenerative Diseases (Deutsches Zentrum für Neurodegenerative Erkrankungen, DZNE), Sigmund-Freud-Straße 27, 53175 Bonn, Germany

## Abstract

This data article provides raw auditory evoked brainstem responses (ABRs) from controls and Ca_v_2.3 transgenics, i.e. heterozygous Ca_v_2.3^+/-^ and Ca_v_2.3^-/-^ null mutants. Gender specific ABR recordings were performed in age-matched animals under ketamine/xylazine narcosis. Data presented here include ABRs upon both click and tone burst presentation in the increasing SPL mode using a commercially available ABR setup from Tucker Davis Technologies Inc. (TDT, USA). Detailed information is provided for the sound attenuating cubicle, electrical shielding, electrode parameters, stimulus characteristics and architecture, sampling rate, filtering processes and ABR protocol application during the course of data acquisition and recording. The later are important for subsequent analysis of click and tone burst related hearing thresholds, amplitude growth function and peak latencies. Raw data are available at MENDELEY DATA, DIO: 〈DOI:10.17632/g6ygz2spzx.1〉, URL: 〈https://data.mendeley.com/datasets/g6ygz2spzx/1〉).

## Specifications table

**Table:** 

Subject area	Biology
More specific subject area	Murine auditory brainstem responses (ABRs)
Type of data	External text (txt.) file
How data was acquired	ABR set up (TDT)
Data format	Raw data
Experimental factors	Female and male Ca_v_2.3^+/+^, Ca_v_2.3^+/-^ and Ca_v_2.3^-/-^ mice were anesthetized using ketamine/xylazine. Subsequently, click and tone-burst evoked ABRs were recorded using a commercially available ABR setup from TDT.
Experimental features	ABR recordings from age-matched female and male Ca_v_2.3^+/+^, Ca_v_2.3^+/-^ and Ca_v_2.3^-/-^ mice.
Data source location	Bonn, Germany
Data accessibility	Data is available at (MENDELEY DATA, DIO: 〈DOI:10.17632/g6ygz2spzx.1〉, URL: 〈https://data.mendeley.com/datasets/g6ygz2spzx/1〉)

## Value of the data

• These data provide resources for investigation of sex-specific differences in auditory information processing in age-matched controls (Ca_v_2.3^+/+^), heterozygous (Ca_v_2.3^+/-^) and homozygous Ca_v_2.3 null mutants (Ca_v_2.3^-/-^) based on click and tone burst evoked ABRs.
• This data collection provides basis for analysis of click and tone-burst related hearing thresholds, amplitude growth function, peak latency analysis etc. via manual or automated tools.
• Data is provided in standardized format to ease access and use for multiple purposes.
• The publication of this dataset will enable users to benchmark their results for comparison with related auditory data on other voltage-gated Ca^2+^ channels and to postulate novel hypotheses on the role of Ca_v_2.3 in auditory processing.

## 1 Data

Ca_v_2.3^+/+^, Ca_v_2.3^+/-^ and Ca_v_2.3^-/-^ mice from both genders were anesthetized using ketamine/xylazine. Following injection narcosis, click and tone-burst evoked ABRs were recorded using a standard ABR setup (TDT). As the TDT raw data file format (.arf) requires TDT specific software (see Section 2.3 below) which is not freely available, raw data were exported as text file (.txt) format and are accessible at (MENDELEY DATA, DIO: 〈DOI:10.17632/g6ygz2spzx.1〉, URL: 〈https://data.mendeley.com/datasets/g6ygz2spzx/1〉).

## 2 Experimental design, materials and methods

### 2.1 Experimental animals

All experimental genotypes (Ca_v_2.3^+/+^, Ca_v_2.3^+/-^, Ca_v_2.3^-/-^) were generated via heterozygous Ca_v_2.3^+/-^ embryos (kindly provided by Richard j. Miller, Department of Neurobiology, Pharmacology and Physiology, The University of Chicago, Chicago, IL, [1]) which were re-derived with C57BL/6J mice and maintained with random intra-strain mating [2]. A neo-URA3 cassette was used to replace the exons 4–8 of Cacna1e via homologous recombination (Mouse Genome Informatics; MGI Ref. ID J: 66144).

For subsequent ABR recordings, Ca_v_2.3^+/+^ controls, heterozygous Ca_v_2.3^+/-^ and homozygous null mutant Ca_v_2.3^-/-^ mice (58 animals in total, aged 141.29 ± 0.39 days (~ 20 wks)) from both age-matched genders were used with the following characteristics: Males: Ca_v_2.3^+/+^: *n* = 9 (♂), weight 32.72 ± 1.80 g; Ca_v_2.3^+/-^: *n* = 9 (♂), weight 31.64 ± 1.12 g; Ca_v_2.3^-/-^: *n* = 10 (♂), weight 31.10 ± 0.89 g. Females: Ca_v_2.3^+/+^: *n* = 9 (♀), weight 25.41 ± 0.57 g; Ca_v_2.3^+/-^: *n* = 10 (♀), weight 25.42 ± 1.36 g; Ca_v_2.3^-/-^: *n* = 11 (♀), weight 27.00 ± 0.43 g.

Experimental animals were housed in groups of 3–4 in clear Macrolon cages type II with *ad libitum* access to standard food pellets and drinking water. Mice were maintained at a temperature of 21 ± 2 °C, 50–60% relative humidity, and on a conventional 12 h light/dark cycle with the light cycle starting at 05:00 AM using ventilated cabinets (Type Uniprotect, Bioscape). The animals were strictly acclimatized to this circadian pattern prior to subsequent experimentation. All animal procedures were conducted according to the Guidelines of the German Council on Animal Care and all protocols were approved by the Local Institutional and National Committee on Animal Care (Landesamt für Natur, Umwelt und Verbraucherschutz, State Office of North Rhine-Westphalia, Department of Nature, Environment and Consumerism, LANUV NRW, Germany). The authors further certify that all animal experimentation was performed in accordance with the European Communities Council Directive of November 24, 1986 (86/609/EEC). Specific effort was made to minimize the number of animals used and their suffering.

### 2.2 Mouse anesthesia

Prior to ABR recordings, animals were anesthetized using ketamine (100 mg/kg body weight i.p., Ketanest^®^ S, 25 mg/ml Pfizer, Germany) and xylazine (10 mg/kg body weight i.p., Rompun^®^ 2%, Bayer HealthCare, Germany) and positioned inside a sound attenuating cubicle (ENV-018V, Med Association Inc., USA) lined with an acoustical foam. In addition, the entire sound attenuating cubicle was covered by a Faraday cage (stainless steel, 2 mm thickness, 1 cm mesh size) to protect ABR recordings from electrical noise. To maintain body core temperature, animals were placed on a homeothermic heating blanked (ThermoLux^®^, Witte + Sutor, Germany; TSE^®^, Bad Homburg, Germany) inside the attenuating cubicle. To protect from corneal desiccation, eyes were covered with an eye ointment (5% Dexpanthenol, Bepanthen^®^, Bayer Vital GmbH, Germany) [2].

### 2.3 ABR recording procedure

For recording of monaural bioelectrical auditory potentials, subdermal stainless steel electrodes (27GA 12 mm, Rochester Electro-Medical, USA) were inserted at the vertex, axial the pinnae (positive (+) electrode) and ventrolateral of the right pinna (negative (-) electrode). The ground electrode was positioned at the hip of the animal. To verify proper electrode positioning/conductivity, impedance measurements of all electrodes (< 5 kΩ) were carried out prior to each ABR recording [2].

All ABR recordings were performed under free field conditions using a single loudspeaker (MF1 Multi-Function Speaker, Tucker-Davis Technologies Inc., TDT, USA) which was positioned 10 cm opposite to the rostrum of the animals (loudspeaker leading edge perpendicular to the mouse interaural axis).

The SigGenRZ software (Tucker-Davis Technologies Inc., TDT, USA) was utilized to program stimulus protocols for both click and tone bursts. The bioelectrical ABR signals recorded from the subdermal electrodes were transferred to a head stage (RA4LI, Tucker-Davis Technologies Inc., TDT, USA) and forwarded to the preamplifier (RA4PA, Tucker-Davis Technologies Inc., TDT, USA) with 20-fold amplification.

The ABR equipment e.g. loudspeaker control, acoustic stimulus presentation (clicks and tone bursts), ABR acquisition, ABR processing, averaging and data management were further coordinated using the RZ6 Multi I/O Processor system and BioSigRZ software (both Tucker-Davis Technologies Inc., TDT, USA).

Note that ABR data acquisition was performed at a sampling rate of 24.4 kHz and signals were bandpass filtered (low pass 5 kHz, high pass 300 Hz) using a 6-pole Butterworth filter. Importantly, the individual ABR data acquisition time (recording signal architecture) was 25 ms starting with a 5 ms baseline period (preABR baseline), followed by a 10 ms ABR section triggered by the individual acoustic click or tone burst stimulus onset and another, final 10 ms baseline (postABR baseline) [2].

The two types of acoustic stimuli were applied for ABR recordings in Ca_v_2.3^+/+^, Ca_v_2.3^+/-^ and Ca_v_2.3^-/-^ mice using the SigGenRZ software (Tucker-Davis Technologies Inc., TDT, USA) and applied via the TDT BioSigRZ platform. The click stimulus entity was an electrical white noise burst of 100 µs duration, with alternating polarity (switching between condensation and rarefaction) and substantial energy in the 0.8–43 kHz range. The tone burst stimulus entity was a 4.5 ms tone burst (transient sinusoidal plus) of alternating polarity with Hann envelope rise and fall times of 1.5 ms duration. The frequency range covered 1–42 kHz in 6 kHz steps. All acoustic stimuli were repeated 300 times at a rate of 20 Hz for averaging.

For ABR threshold recordings, sound pressure levels (SPL) were increased in 5 dB steps for clicks and 10 dB steps for tone bursts, starting from 0 dB up to 90 dB (increasing SPL mode). Sound pressure levels for tone bursts within the range of 1–42 kHz were calibrated each day prior to recording using a microphone (378C01, PCB Pieztronics Inc., N.Y., U.S.A) connected to a preamplifier (480C02, PCB Pieztronics Inc) and the RZ6 Multi I/O Processor system (Tucker-Davis Technologies Inc, TDT, USA). The microphone was placed inside the sound attenuating cubicle and connected to an oscilloscope (DPO3012, Tektronix^TM^, USA) to monitor and confirm the spectrum of sound stimuli using online Fast Fourier Transformation (FFT) [2].
